# Surf and Turf: Mechanism of Enhanced Virus Spread During Poxvirus Infection

**DOI:** 10.3390/v2051050

**Published:** 2010-04-28

**Authors:** Richard C. Condit

**Affiliations:** Department of Molecular Genetics & Microbiology, University of Florida, P.O. Box 100266, Gainesville, FL 32610, USA; E-Mail: condit@mgm.ufl.edu; Tel.: +1 352 273-9523; Fax: +1 352 273-8905

## Abstract

Commentary on Doceul, V.; Hollinshead, M.; van der Linden, L.; Smith, G.L. Repulsion of superinfecting virions: a mechanism for rapid virus spread. *Science* **2010**, *327*, 873–876.

Often the simplest observation inspires the most profound insight. Such is the case with a recent paper from Doceul *et al.* in the February 12 issue of *Science* [1]. The simple observation is that vaccinia virus plaques grow faster than can be accounted for by the replication cycle of the virus. The insight, after a good deal of novel experimentation, is that virus is surfing on a wave of infectivity at the leading edge of a growing plaque, turfed from cells that are infected but not yet producing virus in order to access virgin cells. The observations represent a fresh perspective on the fundamentals of virus replication and at the same time offer opportunities for advances in antiviral therapy.

Vaccinia virus, the prototypical poxvirus, is the virus that was used as a live vaccine for the eradication of smallpox [2]. Poxviruses are large (200 genes) DNA-containing viruses that carry out their entire replication cycle in the cell cytoplasm, and thus must encode all the enzymes required for virus mRNA synthesis and modification and for viral DNA replication. Despite eradication of smallpox, interest in poxviruses persists for several reasons: poxviruses have long served as models for fundamental studies of RNA and DNA metabolism; recombinant poxviruses provide robust vaccine and expression vectors; poxviruses have considerable potential as oncolytic agents; poxviruses are masters of immune evasion and thus provide significant insight into the immune response; smallpox is high on the list of potential weapons of bioterror. Poxviruses are also still full of surprises and insights into basic virology, as the Doceul *et al.* paper reveals.

The vaccinia virion exists in three infectious forms, called IMV (intracellular mature virus), CEV (cell associated enveloped virus) and EEV (extracellular enveloped virus) [3] (Figure 1). IMV contains a single lipid bilayer membrane that is fabricated within cytoplasmic virus replication factories using a novel process that is incompletely understood. As the name implies, IMV accumulate within cells and are released only by cell lysis. IMV are extremely stable in the environment and are thought to enhance transmission of infection from host to host. CEV and EEV each contain two membranes, the original IMV membrane plus one additional outer Golgi-derived membrane. CEV and EEV are produced from IMV via an intracellular intermediate called IEV (intracellular enveloped virus). IEV are formed when IMV bud through or are wrapped by Golgi-derived cisternae, thus acquiring two membranes in addition to the original IMV membrane. IEV are then transported to the undersurface of the plasma membrane where the outermost membrane fuses with the plasma membrane to exocytose a double membraned particle. During the time that this particle remains attached to the cell it is called CEV; once free from the cell it is called EEV. The outer Golgi-derived membrane of CEV/EEV contains seven viral proteins that contribute to CEV/EEV assembly and function. It has long been recognized that CEV/EEV are important for cell to cell and tissue to tissue spread within a host, however the mechanisms governing this spread are only partly understood.

A peculiar feature of vaccinia infection is the formation specialized actin-containing filamentous projections, called “actin tails”, that are implicated in transmission of CEV/EEV from cell to cell [4,5]. Actin tails consist of bundles of actin filaments that form at the point of attachment of CEV to the cell surface, growing to produce a long projections with CEV perched at the tip (Figure 2). Actin tails seem to promote spread of virus from cell to cell since mutation of any of six of the seven CEV/EEV membrane proteins abrogates actin tail formation and results in formation of smaller than normal plaques without affecting IMV formation [3].

Doceul *et al.* measure the rate of growth of vaccinia plaques using time lapse video microscopy of cultured infected cells. These stunning videos are destined to become instant classics and are a must for any basic virology lecture. As noted above, the rate of growth of the plaques significantly exceeds what would be predicted based on the known time course of vaccinia infection. Actin tails are implicated in the rapid plaque growth because virus mutants in actin tail formation form smaller plaques whose growth rate is consistent with the kinetics of virus replication. Additional insight into the rapid spread is provided by videos of plaque formation following infection by a virus that encodes a GFP-tagged virion structural protein expressed specifically late during infection. As expected, green cytoplasmic virus factories are absent from infected cells at the leading edge of a plaque and only appear further toward the center of the plaque, where the infection has entered the late phase and virion assembly is underway. Close inspection of these plaques however reveals CEV-tipped actin tails decorating infected cells that lack green factories at the leading edge of the plaque. Since the cells lacking green factories could not have produced the attached CEV, this observation implies that CEV produced from neighboring cells late during infection are somehow capable of inducing acting tail formation when they come in contact with cells still in the early stage of infection. The authors then show that two CEV/EEV membrane proteins, A33 and A36, that are normally expressed early during virus infection, can promote CEV-induced actin tail formation when expressed in cells outside the context of a vaccinia infection using lentivirus vectors. These results need to be understood in the context of one additional historical observation, namely, that vaccinia infection induces superinfection exclusion [6–8]. Exclusion of IMV is established early during vaccinia infection and may involve a complex of the viral proteins K2 and A56, which are expressed throughout infection and transported to the cell surface. Exclusion of CEV and EEV has not been investigated directly, however Doceul *et al.* observe CEV contacting the same infected cell multiple times and inducing sequential actin tails, an apparent manifestation of superinfection exclusion. In summary, the results show that CEV/EEV are actively repelled from infected cells via induction of actin filaments until they finally encounter an uninfected cell in which a new infection can be established. Thus the combination of actin tail formation and superinfection exclusion have the effect of economizing on the usage of new virions and enhancing spread of the infection.

These experiments have profound significance for both viral pathogenesis and antiviral therapy. As one might predict, virus mutants in A33R and A36R are avirulent in a mouse model of infection as are mutants in other CEV/EEV membrane proteins [1,9,10], thus virus genes affecting spread may be considered virulence factors. This implies that drugs which target spread may prove to be effective antivirals. Indeed the most promising new poxvirus antiviral, ST246, targets the CEV/EEV membrane protein F13, abrogating CEV/EEV formation and preventing virus spread during infection [11]. Numerous other viruses induce superinfection exclusion, and it would be no surprise to learn that other viruses have evolved sophisticated mechanisms for enhancing spread as well. Thus poxviruses may once again play a pioneering role in advancing understanding virus replication and pathogenesis.

## Figures and Tables

**Figure 1.**
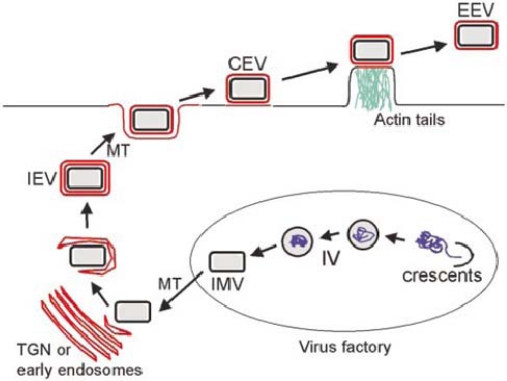
Overview of VV assembly and egress. In virus factories, viral membrane crescents encapsidate viral proteins and DNA to from immature virions (IV) which morph into IMV. IMV move on microtubules (MT) to the Golgi network where they are acquire a double membrane to form IEV. IEV migrate to the cell surface where the outermost IEV membrane fuses with the plasma membrane to form CEV. CEV induce actin tail formation to drive the virion away from the cell. CEV may also be released to form EEV. From [3].

**Figure 2.**
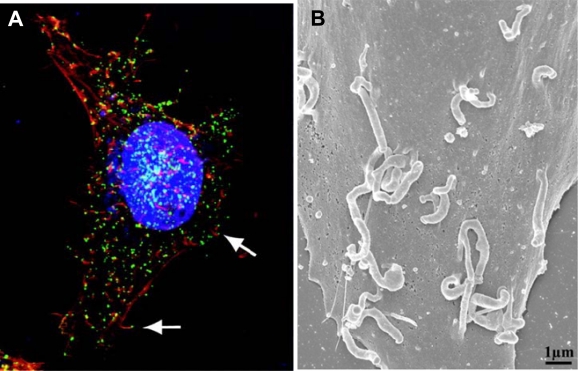
**(A)** CEV tipped actin tails imaged by confocal microscopy. Nuclei and cytoplasmic virus factories are stained blue, actin is stained red and CEV are stained green. The arrows point to CEV at the tips of actin tails. From [12]. **(B)** Scanning electron micrograph of a vaccinia infected cell showing CEV tipped actin tails. From [9].
